# The Relations of Medical Examining Boards to the State, to the Schools and to Each Other

**Published:** 1896-07

**Authors:** William Warren Potter

**Affiliations:** Buffalo, N. Y., Examiner in Obstetrics, New York State Medical Examining and Licensing Board. 284 Franklin Street


### THE RELATIONS OF MEDICAL EXAMINING BOARDS TO THE STATE, TO THE SCHOOLS AND TO EACH OTHER.

By WILLIAM WARREN POTTER, M. D., Buffalo, N. Y.
Examiner in Obstetrics, New York State Medical Examining and Licensing Board.

*Presidential Address before the National Confederation of State Medical Examining and Licensing Boards, at its sixth annual meeting, held at Atlanta, Ga., May 4, 1896.*

IN ASSUMING the duties of this chair my mind instinctively reverts to its first occupant, who as you all very well know was the founder and organiser of this confederation, a pioneer in medical educational reform in this country, an earnest and forceful advocate of every reasonable method of advancing the standard in the United States. Though he was our leader he was also our companion and friend, and if he were yet living he would most likely continue to occupy the chair. To be regarded as worthy of the succession is an honor that I fully appreciate. Let us always keep green the memory of John H. Rauch.

### INTRODUCTION.

There is food for much thought in undertaking to direct the affairs of this organisation. It is a body unique among medical societies. It is without a parallel in medical history and has not even a complete record of its own work. This fault, however, I hope will not happen in the future. The chief function of the confederation is to discuss questions relating to medical examinations for license, but it cannot bind its members to follow its recommendations ; nor does it so desire. In order to make the work of such a body effective all adhesion to it must be voluntary in character, not coercive.

There are, however, many collateral questions to consider if not to settle, but everything must be done with circumspection. We are on the threshold of new conditions. We may, therefore, with great propriety adhere to, if not adopt for our guidance, the ancient maxim "*Festina lente.*" We are without traditions, without precedents, without heredity. There is much wisdom, then, in making haste slowly that we may proceed to accomplish the end sought all the more surely.

### POINTS OF AGREEMENT.

Of one thing I assume we may feel sure—namely, that there are no differences of opinion among physicians even of the several so-called systems of practice recognised by law — intelligent physicians — as to the propriety, even the necessity, of improved methods in medical education and increased standards of acquirements for entrance to the profession of medicine. It has taken considerable time and occasioned some severe contention to reach even this first landing-stage, but I think we are all agreed at last on this point and I also believe the agreement will endure.

As to just what these reforms ought to be may not yet have been determined with unanimity, but I shall assume that all of the class named are of one opinion on some that are necessary. One of these is that there must be a better standard of academic preliminaries; another that ought to bring us together is that four years is little time enough to be devoted to medical collegiate training; and still another we ought to be united upon is, that an examination of each graduated physician by the state, separate and apart from the schools, is a necessary condition precedent to obtain a license to practise. These are three cardinal principles it seems to me on which reforms should be grounded, and it ought to be possible for all physicians to unite in an effort to secure legal enactments in all the states establishing this triad of essential principles.

### PRELIMINARY REQUIREMENTS.

In regard to preliminary requirements for matriculation in a medical school, there may be some difficulty in establishing at once in every state a standard as high as should be, but it seems as if we might agree upon a minimum below which no person may enter upon the study of medicine. It is as requisite for a physician to possess an education as it is for him to be a gentleman.

For myself I would insist that a high school diploma ought to be regarded as an index of the lowest limit of literary attainment for the novitiate. An entrance examination should be required of all who do not possess such a diploma or its equivalent, such examination to be conducted independently of the medical colleges. As I would have the graduate examined by the state for license separately and apart from the college, so I would have the candidate for admission to the study of medicine also examined by the state to determine his qualifications to enter upon that important duty. In other words, the state has the right to determine the qualifications of all who enter upon the study of medicine, as well as those of all who after graduation propose to enter upon the practice thereof, and I am in favor of its exercising both functions.

Not until after four years' study in a recognised medical college and the receipt of a diploma therefrom should a physician become eligible to license by the state, and then only upon due examination by its medical examiners, none of whom should be medical college teachers. It has been claimed by some teachers that state examiners are neither competent to propound proper questions nor to mark them at their true value[1], but I need not absorb valuable time in the discussion of a question that seems to be already definitely settled in the affirmative.

### LIMITATIONS OF STATE CONTROL.

Thus the state again steps in and exercises her right to determine as to the adequacy of the training a physician has received in the schools, and it issues a license to practise only to those found qualified. Hence, there are three stages of preparation for medical practice : first, a preliminary training that shall at least equal that of a high school graduate ; second, four years' study in a legally incorporated medical college ; and, third, examination and license by a state board, none of whose members are teachers in a medical college.

In two of these stages the state plays an active part — namely, in the first and the last. The state must determine as to the competency of those who enter upon the study of medicine and also the qualifications of those who are admitted to practise. But this is the limit of the state's authority ; beyond this it may not interfere : that is, it is not competent for the state to interpose its authority during the medical collegiate life of the student. Here the teachers are supreme and must be left untrammeled. They must be allowed perfect freedom to teach in their own way. Their function is to teach their students so well that they may pass any of the state boards, and they may be trusted to do it, else they will suffer not only the humiliation of rejected candidates but their classes will diminish in size, and, except in the endowed schools, their incomes will correspondingly shrink. Students will not be slow to detect this nor to select the medical college that will prepare them with certainty to obtain the state license.

While, therefore, the state may not interfere, as we have said, during the four years of medical college training, it yet exercises a watchful care in granting charters to medical colleges, issuing them only where the applicants are shown to be teachers of undoubted competence and men of integrity. A state must also be sure that the number of schools within its boundaries is not too large.[15] Moreover, it should specify the essentials to be maintained, such as laboratories for teaching chemistry, physiology, anatomy, bacteriology and the like; also that clinical teaching must be pursued and that hygiene, preventive and state medicine, and lastly, clinical midwifery must be taught,—the latter to the extent that each graduate must have attended at least six cases of labor.

### SHALL THE EXAMINATIONS BE DIVIDED?

It has been held by some that it would be better to divide the state examinations, and at the end of the second year let students come up before the state board for examination in the so-called fundamental branches—anatomy, physiology and chemistry. This would unload the mind, say the advocates of this plan, of a great anxiety, and thus better prepare students to cope with the practical subjects — surgery, obstetrics, practice, materia medica, pathology and hygiene. It is doubted if the state can legally lay its hands on a student after he has passed the entrance door of a medical college, even for the purpose of compelling a division of the examination as has been proposed. The most it could do in the premises would be to *permit* him to take an intermediate pass examination if he so desired; but unless this were made compulsory it would doubtless fail to commend itself to any considerable number and so fall short of its purpose. Besides, it could not be made to apply to foreigners or to students from other states. Taken altogether this plan may be not only impracticable but most likely impossible to establish. Moreover, the theory of the state examination is that it is a post-graduate scrutiny and not an undergraduate test.

### A QUARANTINE AGAINST THE UNQUALIFIED.

The number of states that in some manner supervise the practice of medicine, now proportionately large[2], is constantly increasing. Ohio is the latest convert and it is a genuine pleasure to welcome her to the fold. Our friends in that great common wealth have occupied a trying position for some years, but at last have scored a good start and under the discreet leadership of able men who are handling the reins will reach the goal. Under the ratio of progress now making it will not be long before all the states will in some form or other establish a quarantine against unqualified doctors. But the great desideratum is to persuade each and every state to enact laws that shall require separate examination after graduation in order to obtain license to practise. Until each and every state shall deny the right to practise, except through license obtained on due examination and after graduation from a legally incorporated medical school requiring academic preliminaries and a four years' course, the struggle must continue.

By a law of the state of New York, passed March 21, 1896, it is provided that all candidates for admission to the state examination must have studied medicine not less than four full *school* years *of at least nine months each*, including four satisfactory courses *of at least six months each*, in four different calendar years in a medical school registered as maintaining at the time a satisfactory standard.[1] This section is to take effect January 1, 1898. It is further provided in the same act that New York medical schools and New York medical students shall not be discriminated against by the registration of any medical school out of the state, whose minimum graduation standard is less than that fixed by statute for New York medical schools.

It will be readily understood, in the light of the foregoing extracts from the statute, how impossible it is for New York to recognise the licenses of any state that maintains a standard lower in any respect than its own. Nevertheless, whenever any state shall demand the same preliminaries, an equal collegiate training, together with a commensurately high final examination, certainly it then will be a pleasure for New York to establish reciprocity with it.

It is not my purpose to forestall the discussion on this question that is to come up later at this meeting, but it is not easily possible to omit some mention of this important subject, inasmuch as it is just now very much in evidence all over the country. New York has been criticised[3] by other states for not recognising their licenses, but her position in refusing to indorse the licenses from states in which the standard is lower must be conceded to be impregnable. To recognise licenses from states in which the requirements are less would be to deal unjustly with her own citizens, and to yield advantages over them to those coming from without her boundaries.

### A NATIONAL LICENSING BOARD.

Another question that has been raised in certain quarters is the propriety of creating a national licensing board. Dr. C. E. Far-

1. The italics in this sentence are in the original bill.

num, of San Francisco, I believe, has suggested that such a board be detailed from the medical corps of the army and navy[4]; and that the licensees of this board receive a degree or title similar to the English F. R. C. S., such, for instance, as "Fellow in American Medicine and Surgery."[1]

It seems to me that a national examining or licensing board is not only unnecessary but quite impracticable at present, and I doubt if it ever becomes a measure of expediency. Up to the present time, congress has very wisely interpreted the constitution to relegate police regulations to the several states and has declined to interfere in such matters. The power of congress to create such a board, even were it so disposed, is seriously questioned by well-informed men both of the legal and medical professions. Moreover, the vast machinery required to carry on the work of such a board in territory of such wide domain as ours, would at once suggest an adequate reason for condemning the scheme even if there were no other. As to the proposition of our friend from the Pacific coast to create a board from the public services that shall have power to confer a special degree, it would appear to most of us, I am sure, that our essential need now is not more degrees but better education. Furthermore, we need not superimpose this Herculean task upon our good friends of the medical corps of the army and navy in addition to their already burdensome duties and responsibilities.

Let us wait then until the states themselves — those that have not already done so — advance their standards to the line of requiring preliminary education equal to a high school course, four years' medical collegiate training and a separate state examination for license after graduation. When this is done in our fifty-three states and territories[5] it will be quite time enough to delegate to the general government the duties and powers now exercised by each state in this matter.

### A UNIFORM MINIMUM STANDARD OF REQUIREMENTS.

While, therefore, it is practically impossible at present under existing laws to establish a uniform minimum standard of requirements in all the states, there is yet hope that year by year this desideratum may be more nearly approached until ultimately it shall be attained. Amendments to imperfect laws must be fostered and states without practice laws must be encouraged to enact them, to

1. In a personal letter Dr. F. claims to suggest an examining and not a licensing board, but the difference is not material in this relation.

the end that their citizens may not be discriminated against by the other states.

It has been suggested that a committee of supervision might be created, by the examining boards of those states that require examination after graduation for license forming a syndicate, and each appointing a representative to such a central body. The duties of this committee merely would be to formulate a uniform standard for the guidance of the state boards represented in the committee. As fast as states enacted or amended their laws to meet the established grade of requirements, their boards would be entitled to representation on this central committee; so, whereas, only a small committee at first would be created, in the end it would comprise a membership equal to the whole number of states and territories.

### RECIPROCITY OF LICENSURE.

Between the states represented in this committee, of course, there would be established a reciprocity of licensure. But let us pause for a moment to inquire as to the necessity or desirability of reciprocity. I confess that I am not among the number that regard reciprocity as of great immediate importance. Whenever a sufficient number of states shall advance their standards to a common minimum level of requirements, and these shall demand first, a high school course as an entrance minimum; second, four years' medical collegiate training as a condition for a medical degree; and, third, a separate post-graduate examination for license—when, I repeat, these shall be the requirements for the practice of medi cine in at least twenty states, it will be quite time enough for those commonwealths to establish reciprocity among themselves. Until then let the migratory physician pay the penalty of his itinerancy by taking the examination of his new-sought state. If he is well equipped he will be perfectly willing to do so; if not then let him seek another occupation. Whenever twenty states shall form such a reciprocal syndicate it will not then be long before the others in self-protection will advance their standards to the minimum these shall have established.

### THE STATE SHOULD EMPLOY ONLY LICENSED PHYSICIANS.

It is impossible to create new conditions such as we are entering upon without meting out hardship to some individuals. All border line students and young physicians naturally feel themselves aggrieved, and they vehemently demand special rulings or statutes to fit each particular case. But we must begin somewhere to draw the line between the old way and the new, and the few ought to yield gracefully to the many. The particular good must not stand in the way of the general good.

The state is ever jealous of her rights and of the welfare of her citizens. She is particularly so of their good health, which means economy. She has assumed to decide who shall and who shall not minister to the sick and injured and she especially has determined to administer the laws of prevention with a constantly increasing rigidity. Her public health officers must be men of education and executive capacity. It will not be long, I trust, before no person will be commissioned in any of these great offices who does not possess a state license. If this were made the rule we would soon hear less complaint in regard to the state examination; while, on the other hand, every spirited young graduate would be anxious to take it. Such action would tend to remove prejudice against the system and would in many ways strengthen the hands of those who are engaged in this reform.

### HOW SHALL FOREIGNERS BE DEALT WITH ?

One of the difficult problems confronted is in dealing with foreigners. These men come to this country in large numbers without knowledge of our language, where they are told that everything is as free as air, hence they expect to be admitted to practice at once without let or hindrance. Finding a state examination necessary they plead poverty and demand leniency because of their imperfect knowledge of the English tongue. The question presented may be formulated about as follows: "Shall one rule be established for our own countrymen, and another less rigid for these strangers?" I trust not and I hope the answer will be a unanimous negative. The injustice of such discrimination against our own citizens is too apparent to admit of argument. I would not make one rule for one class of candidates and another for another class, but I would administer the laws with impartiality, governing all alike.

If one of our fellow-citizens should present such examination papers to a foreign board as these men generally offer to most of ours, he would be denied even the semblance of a hearing. His application would be dismissed without ceremony. Let it be remembered in connection with this that the country is not suffering for the want of doctors, and can wait without material injury until these men shall master the English language and otherwise conform to our rules,—until they can place themselves on the same footing in every respect with our own countrymen. When they present themselves in a clear identity, with a legal diploma properly authenticated, and take our examination successfully, then we will gladly issue to them licenses to practise, but they should be made to understand at once that they can obtain them in no other way. This question is attracting the attention of medical journals in different sections of the country and has lately been discussed by one[6] in a most decided and uncompromising manner.

### BOARDS NOT ANTAGONISTIC TO THE COLLEGES.

It has been asserted in certain quarters that state boards are antagonistic to colleges, that they are setting up standards of their own, and that their rules are oppressive to the schools. Nothing, in my view, could be further from the truth. The real facts are that the boards and the colleges are working along parallel lines to accomplish the same end — namely, an improvement in the quality of physicians admitted to practice in the United States. Moreover, there is a harmony of action between them that is remarkable, considering the radical changes that have necessarily been wrought in methods of teaching as a result of the practice laws. If the schools in many instances have waited for mandatory laws to raise their standards and increase their years of study, they must not complain that the rank and file of a great profession has risen in its might, and through its constituted state medical societies demanded laws of the several state legislatures that shall advance the cause of higher medical education. The examining boards are merely the servants of the people in this matter,—are simply instruments through which their will obtains definite expression.

There can be no antagonism against the schools in this needful reform. They are as much interested in it as are we. We are not enemies, but friends. The college teachers are more than glad to be relieved of much of the detail of educational reform, and thus to be enabled to address themselves solely to clinical, laboratory, recitative and didactic teaching. They prefer not to concern themselves about entrance examinations, thus avoiding the thousand and one questions that are constantly being put by the students on this and kindred subjects relating to detail. The object of the schools is to see to it that young physicians are properly equipped to practise medicine, while the purpose of the boards is to determine that physicians have been adequately instructed to merit a state license. Each has its appropriate place; each serves a distinct purpose.

In a recent conversation a distinguished teacher in one of the first medical schools in the land remarked: "The faculty of which I am a member desires to teach our pupils so well that they may be enabled to pass any examining board,—army, navy or state. If perchance we fail to do so now and then, or if some escape our scrutiny, we are only too glad to have the state board reject such and send them back for further training. We approve of the good work doing by the examining boards, and are glad to have them supervise our methods to the end that incompetent doctors may not be sent out from our college." Words of this character coming from such a distinguished source cannot fail to do good. They express the entire relationship that ought to exist between the schools and the boards. If teachers all felt this way and so expressed themselves whenever occasion presented, it would serve to strengthen the hands of the examiners, increase their usefulness and so benefit the cause of educational progress.

I fear, however, teachers in many instances have engendered in the minds of students a dread of the boards and, by innuendo if not by words, belittled their work.[7] Students are taught in some schools in a way to lead them to suppose that the only object of colleges is to prepare them to successfully compete for the state license. It is forgotten, apparently, that the real aim should be to teach them how to be good physicians. If they would stimulate in their classes a respect for the boards and instruct them that the state license is a parchment to be eagerly sought and highly prized when won, it would tend to create an atmosphere around them that would prove of lasting benefit to the cause. It affords me pleasure, however, to refer in this place to two notable exceptions to the condition I have just delineated. Prof. Hinkel, of Buffalo, devoted his introductory lecture in a college course not long ago to the subject of state examination for license,[8] in which he fully set forth the system in operation in New York, and instructed the students as to the importance of the measure. Prof. Tucker, of Albany, chose as the title of his opening lecture last fall at the Albany medical college, "State control in medicine."[9] He, too, carefully analysed the new system and gave deserved credit to the examining boards. Undoubtedly there have been many similar instances where teachers have taken occasion to speak well of examinations by the state for license, but I need not refer to this subject in greater detail at this time.

### MEDICAL JOURNALS ARE AIDING.

It is a satisfaction to know that the medical periodical press is very nearly unanimous in its support of the principles advocated in this paper. Moreover, it is an active ally that is constantly busy in disseminating information on the subject, and urging on the most efficient and highest order of reform. While it is true that at first many were sceptical, some were lukewarm, and a few were open enemies of the scheme, now it is a pleasure to affirm that there is not a single medical magazine of standing, weekly, monthly or quarterly, between New York and San Francisco and from Maine to Texas,[10] which is not an aggressive coadjutor, while many are propagandists of the faith. These magazines are leaders in thought and moulders of opinion, and under their influence we may hope soon to advance and unify standards, to a point where all states and territories of the Union will have practically established the same requirements for obtaining license to practise.

### A VOICE FROM THE TRANSVAAL.

A strong argument for uniformity of methods and standards throughout the Union is presented, incidentally, in a recent letter addressed to the *Medical Record* by Dr. Gordon Messum, chairman of the Transvaal Government Medical Board. The rule followed there is for the board to register only such physicians as present diplomas which entitle the legitimate holders to practise in the whole of the country where such diploma has been issued, and for which a minimum of four years' medical study is required. The board finds it difficult, writes Dr. Messum, to decide on the merits of the various diplomas issued in the different American states, (is there special wonder ?) and asks information on the following points :

1. Are there state universities or colleges whose degrees are recognised as giving the right to practise throughout the whole of the United States of America ?

2. Can we obtain a list of universities or colleges which demand a four years' course, and can the approximate date be given when such four years' course became compulsory in such colleges ?

3. Is there a list of the universities and colleges whose degrees are recognised by the medical board of the United States of America ?

4. Are there registers of the qualified men in each state, or is there a general register for the whole of the United States of America on which the name of every qualified man can be found?[11]

Here is the South African Republic refusing to admit to practice any physician with less than four years' collegiate medical training, while most states in the Union yet hesitate to establish that minimum. When this confederation has made adequate answer to the voice from the Transvaal it will have accomplished a very essential part of its mission.

### A CONDITION OF UNREST.

The fact must be apparent to everyone who has given the questions arising out of state licensure much thought, that many of the difficulties which we are meeting, some of which I have considered, arise from the fact that we are in the midst of a period of unrest. The doctors are clamorous, legislators are feverish, and students are excited. Nearly everybody having to do with the subject directly or indirectly seems to be uncertain as to opinion and unstable in action. Many appear to be standing on tip-toe, all agog, waiting to hail something newer and stranger than that which went before. It is not unlike the excitement that follows upon the discovery of a new bonanza ; but a period of quietude will soon follow if this confederation assumes its appropriate place and wisely exercises the functions that properly belong to it. It must begin now to take cognisance of our environment and to make suggestions for its betterment.

It is this restless spirit that gives birth to so many incongruous and untimely suggestions. Now it is a proposition to divide the state examination ; then to create a national examining and licensing board ; again, a demand for reciprocity of licensure, and at another time legislatures are appealed to to reduce the barriers to entrance upon the study of medicine, as recently happened in the state of New York. These and other propositions are hurled at us one after another, singly or in groups, with a rapidity that would dismay any but the intrepid, and with an audacity worthy of disciplined ranks. And this before the state boards have fairly formulated their work, or, at any rate, before any tangible results of the system are manifested. But let it be remembered that "thrice is he armed that hath his quarrel just," and so we may take much comfort in the thought that the people, though slow to arouse themselves to reforms, when once determined on a line of policy that is just and needful, are seldom turned aside until their full purpose is accomplished.

### CONCLUSION.

But I detain you too long from the enjoyment of the intellectual feast that awaits you. The recent action of Harvard University in giving notice that a degree will soon be required for an entrance to its medical school,[12] and the statement that the University of Pennsylvania will raise her standard,[13] indicate that a movement has commenced of a most substantial nature. Though there is a vast field before us in which to labor for the advancement of educational standards there is every reason for encouragement from the support that medical teachers, medical journals, educators and many other people of intelligence are affording us.

This organisation ought to be the means of hastening on the work to a considerable degree. An interchange of thoughts and methods through the annual conferences of this body will tend to unify and standardise much of the work that otherwise would be without system or harmony. This body, to borrow the thought of another, should act as a kind of professional clearing house,[14] and I trust it will soon come to be so regarded by all interested in educational progress. Let me also suggest that the *Bulletin of the American Academy of Medicine* be made the medium during the intervals of our meetings for an interchange of thought on all matters of interest to the confederation. It has been made, by formal action, the official journal of this body, and its columns are open to all our members. The American Academy of Medicine and the Association of American Medical Colleges are to be regarded as our allies in the field in which we labor. It is fitting, therefore, that these three bodies, between which there is near kinship, should record their work in the pages of the same magazine, which is a journal devoted especially and exclusively to improvement in medical education.

Finally, let me urge united action by all the friends of medical progress everywhere throughout the land to the end that the education of American physicians may be brought to a standard that shall be high enough at least to make them the peers of their European confrères. The reproach cast upon us through a refusal to recognise our diplomas in Europe cannot be overcome until we rise in our might and determine to wage a relentless war against ignorance, which shall not cease until an American state license to practise medicine is recognised as a passport to good professional society in every civilised country in the world.

REFERENCES.

1. Thos. H. Hawkins, editorial in Denver Med. Times, September 2, 1895.
2. Vide Bull. Am. Acad. Med., Vol. II., No. 4, p. 360, Dec., 1895.
3. Fifth Annual Report Medical Examiners N. J., 1896, p. 5.
4. N. C. Med. Jour., Feb. 5, 1896, p. 5.
5. Bull. Am. Acad. Med., Vol. II., No. 4, p. 360, Dec., 1895.
6. Lancet-Clinic, Cincinnati, April, 1896, p. 441.
7. Am. Med. Compend, Toledo, March 1896, p. 85.
8. Buffalo Med. Jour., Nov., 1894, p. 193.
9. Author's reprint, C. F. Williams, Printer, Albany, N. Y., 1895.
10. Vide Texas Medical News, March, 1896, p. 219.
11. Medical Record, April 11, 1896, p. 533.
12. Boston Med. and Surg. Jour., Jan. 9, 1896, p. 46.
13. Univ. Med. Mag., March, 1896, p. 443.
14. Dr. McIntire, in report of com. on revision of constitution, Bull. Am. Acad. Med., Vol. II., No. 4, p. 351.
15. Ohio has determined lately that colleges shall not be recognised in cities under 50,000 inhabitants.

284 FRANKLIN STREET.

